# The predictive role of toll-like receptor-4 genetic polymorphisms in susceptibility to and prognosis of prostatic hyperplasia

**DOI:** 10.22038/ijbms.2018.33173.7922

**Published:** 2019-01

**Authors:** Yunhua Qiu, Jinzhou Zheng, Jianfeng Yang, Feng Li, Xiqiu Zhou, Xiaoyun Song

**Affiliations:** 1 Department of General Surgery, Pudong Branch of Longhua Hospital, Shanghai University of Traditional Chinese Medicine, Shanghai 200032, P.R. China

**Keywords:** Genetic variation, MDR model, Prognosis, Prostatic hyperplasia, Risk, Toll-like receptor 4

## Abstract

**Objective(s):**

This study was aimed to evaluate whether single nucleotide polymorphisms (SNPs) of TLR4 and common living habits of prostate hyperplasia (BPH) patients would affect the subjects’ risk and prognosis.

**Materials and Methods:**

We totally recruited 501 BPH patients and 964 healthy controls. The patients’ international prostate symptom score (IPSS) and quality of life assessment (QoL) were designated as the prognostic indexes for BPH patients. Altogether 7 SNPs within TLR4 were selected, and the interactions among SNPs and living habits were explained with multi-factor dimensionality reduction (MDR) modeling.

**Results:**

The mutant alleles of rs10983755 (G>A) and rs1927907 (G>A) tended to put on risk of BPH, yet the wide alleles of rs4986791 (C>T) and rs115336889 (G>C) were associated with incremental susceptibility to BPH (*P*<0.05). The rs10983755 (GA) and rs1927907 (GA) were suggested as the marker of non-aggressive BPH, whereas rs4986791 (TT) could symbolize aggressive BPH (*P*<0.05). The homozygotes of rs4986791 (TT) and rs115336889 (CC) could improve the IPSS change, and rs115336889 (CC) was also correlated with more obviously ameliorated Qol change (*P*<0.05). Finally, MDR modeling suggested that rs4986791 (TT) and rs115336889 (GG) shaped the genotyping combination featured by the lowest risk of BPH when smoking or drinking history was also evaluated.

**Conclusion:**

The SNPs situated within TLR4 were potent candidates for predicting risk and prognosis of BPH patients, and their interactions within environmental parameters also helped to develop effective strategies for preventing and treating BPH.

## Introduction

Benign prostatic hyperplasia (BPH), one major cause of voiding disorder among middle-aged and elderly males, was accompanied by increasing incidence with age (1). Usually BPH firstly occurred among the population aged > 40 years old, and its morbidity rate could rise up to 83% in the patients’ 80s (2). According to statistics, approximately 50% of patients with histologically diagnosed BPH displayed moderate-to-severe lower urinary tract symptoms (LUTS), which brought serious interference to patient’s life quality (1). 

Compelling hypothesis proposed that lipopolysaccha-

ride (LPS)/toll-like receptor (TLR)-4 signaling could inhibit functioning of bone morphogenic protein (BMP) and activin membrane-bound inhibitor (BAMBI), thereby facilitating epithelial-mesenchymal transition (EMT) underlying development of BPH (3). The TLR4 herein belonged to TLR family, which was a vital pathogen-recognition receptor involved with innate immunity and played an indispensable part in guiding the immunity process of pathogens (4-6). Moreover, TLR4, located at 9q32-33 and with a total length of 3 811 bp, was widely distributed within immune cells, including T lymphocytes, B lymphocytes, leukocytes and monocyte macrophages (6). The TLR4 also could cooperate with TLR2 and receptor for advanced glycation end products (RAGE) to activate NF-kB via integration with high mobility group B1 (HMGB1), which finally generated positive feedback to inflammatory reactions (7).

Of note, single nucleotide polymorphisms (SNPs) were DNA sequence polymorphisms caused by variations in single nucleotides at the genomic level, accounting for more than 90% of all known polymorphisms. Dysfunctioning of essential SNPs usually could contribute to elevated prevalence of disorders. For instance, it was reported that three SNPs of TLR4 (i.e. rsl927914, rsl927911, rs2149356) were associated with altered risk of coronary artery disease and atherosclerotic plaque calcification (8). In addition, the site of rs2149356 can influence susceptibility to type 2 diabetes by adjusting total cholesterol (TC) and high density lipoprotein cholesterol (HDL-C) levels (9). In view of the causal relationship between TLR4 and BPH onset, it was suspected that SNPs within TLR4 could induce higher susceptibility to BPH.

Nonetheless, limited studies have been conducted to explore the correlation between SNPs of TLR4 and risk or prognosis of BPH, let alone the interactive impacts of multiple SNPs on susceptibility to BPH. Furthermore, BPH was a multifactorial disease related to theories like the functions of hormones and non-androgen substances in the testicles, the interactions of mesenchymal-epithelial, growth factors and so on (10). That was to say, this disorder did not follow Mendel’s law of inheritance, and interactions among gene loci readily resulted in complicated high-order interaction (11). In response, this investigation focused on the correlation between SNPs within TLR4 and BPH risk by feat of multifactor dimensionality reduction (MDR) model (12), which might offer evidences for efficacious diagnosis and treatment for BPH.

## Materials and Methods

***Subjects***

Totally 501 hospitalized BPH patients were recruited from July 2013 to June 2016 in Urology at Pudong Branch of Longhua Hospital, Shanghai University of Traditional Chinese Medicine. All specimens were confirmed by histopathology. The diagnostic criteria for BPH patients were verified as: 1) moderate to severe lower urinary tract obstruction symptoms (IPSS score); 2) maximum urinary flow rate were decreased (Qmax was < 5 ml/sec when urine volume was over 150 ml); 3) prostate volume was > 30 cm³; and 4) level of prostate specific antigen (PSA) was < 4 ng/ml. If PSA level was greater than 4 ng/ml, prostate biopsy would be performed to exclude prostate cancer, and PSA changes were meanwhile closely monitored. When PSA continued to rise and prostate cancer could not be diagnosed, the subjects would not be included into this study. Moreover, BPH subjects satisfying the following items would be excluded: 1) they showed urinary obstruction symptoms caused by non-benign prostatic hyperplasia; 2) they showed frequent urination, urgency symptoms caused by urinary tract infections; 3) they suffered from neurogenic bladder; 4) they displayed oliguria as a result of renal failure; 5) they were diagnosed with prostate cancer; 6) they displayed chronic prostatitis with overt LUST symptoms; 7) they were diagnosed with bladder cancer; and 8) they were confirmed with prostate hyperplasia and required surgery.

Among the BPH population, subjects that satisfying one of phenotypes below were included into the non-aggressive group: 1) their symptoms were controlled poorly during medication, and IPSS staging increased by > 2 per year; 2) some BPH complications were observed, including recurrent hematuria, repeated urinary tract infection, acute urinary retention, cystolith and renal impairment (i.e. serum creatinine > 120 μmol/l); and 3) disease progression demanded further operative treatment. Furthermore, other BPH population that met the following standards were arranged into the non-aggressive group: 1) medication could effectively control related symptoms, and the improved IPSS scoring remained stable; 2) complications related with BPH were hardly observed; and 3) operative treatment were unnecessary. Simultaneously, the urologists included 964 healthy controls through investigating their medical history, digital rectal examination and prostate ultrasound. All subjects were informed in advance of protocols of this investigation, and they have signed consents. Moreover, this study was approved by Pudong Branch of Longhua Hospital, Shanghai University of Traditional Chinese Medicine and the Ethics Committee of Pudong Branch of Longhua Hospital, Shanghai University of Traditional Chinese Medicine.

**Figure 1:**
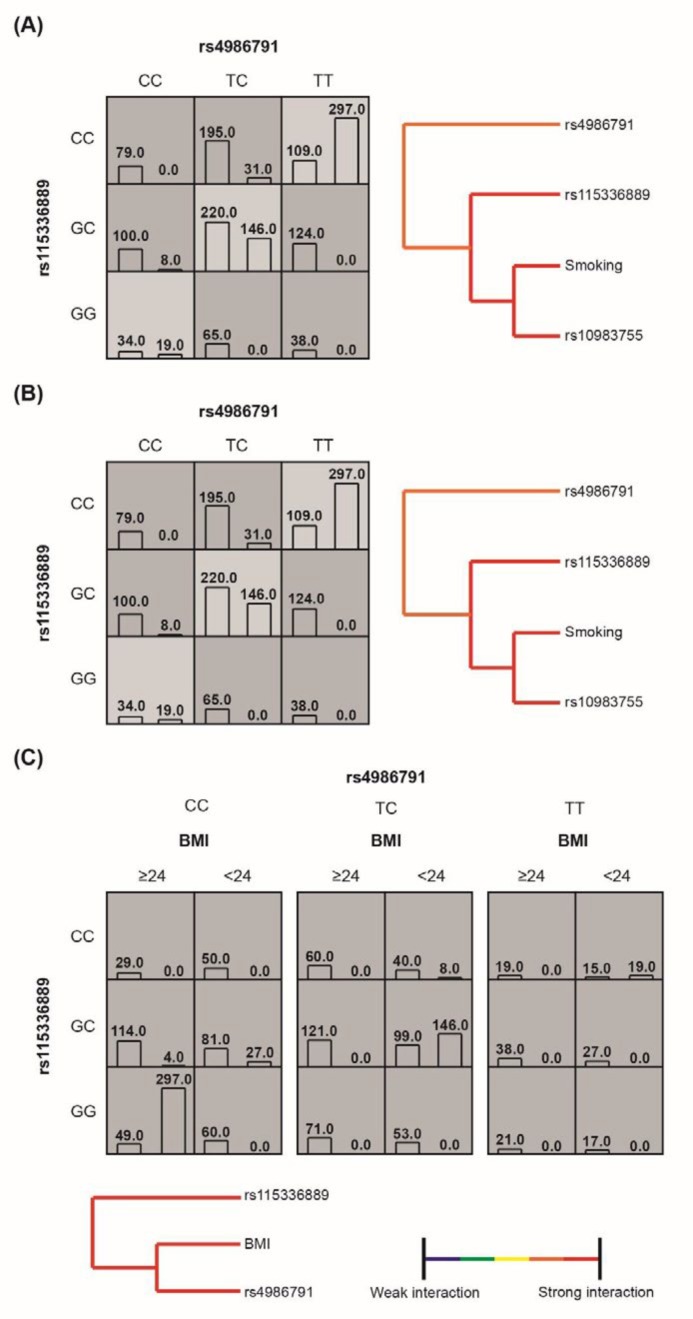
High- and low-risk combinations, hierarchial interaction graphs and interaction dendrograms drawn from establishment of multifactor dimensionality reduction models for inducing benign prostatic hyperplasia among single nucleotide polymorphisms within toll-like receptor-4 and smoking (A), drinking (B) and body mass index (C). The dark blocks were highly risky groups, and the light blocks were lowly risky groups. The vertical bar on the left side symbolized the number of cases, and the vertical bar on the right side symbolized the number of controls. BMI: body mass index

**Table 1:** Comparison of the baseline characteristics between benign prostatic hyperplasia patients and healthy controls

**Clinical characteristics**	**PatientsN=501**	**Healthy controlsN=964**	**t/x** ^2^	***P*** **-** **value**	**OR (95% CI)**
Age	71.43±5.31	70.65±6.43	1.87	0.06	
Disease type					
Aggressive	216	-			
Non-aggressive	285				
Smoking					
Often	372	590			
None/seldom	129	374	24.89	<0.001	1.83 (1.44-2.32)
Alcohol					
Often	343	562			
None/seldom	158	402	14.42	<0.001	1.55 (1.24-1.95)
BMI					
≥24	301	522			
＜24	200	442	4.71	0.03	1.27 (1.02-1.59)
IPSS	18.64±7.51	-			
QoL	3.08±0.92	-			
tPSA (ng/ml)	2.14±1.20	1.22±1.16	14.23	<0.001	
TPV (ml)	34.12±11.08	22.45±13.15	16.98	<0.001	
Qmax (ml/s)	12.85±5.64	25.82±10.40	26	<0.001	
PVR (ml)	23.74±10.13	12.63±7.31	24.07	<0.001	

BMI: body mass index; IPSS: international prostate symptom score; QoL: quality of life assessment; tPSA: total prostate specific antigen; TPV: total prostate volume; PVR: post voiding residue; OR: odds ratio; CI: confidence interval.

**Table 2:** Association of the single nucleotide polymorphisms within toll-like receptor-4 with susceptibility to benign prostatic hyperplasia

**SNP**	**Allele ** **c** **hange**	**Case genotype**			**Control genotype**			**Case allele**		**Control allele**		**Odds ratio**	**Lower limit**	**Upper limit**	***P*** **-** **value**
		**WW**	**WM**	**MM**	**WW**	**WM**	**MM**	**W**	**M**	**W**	**M**				
rs10983755	G>A	361	128	12	613	308	43	850	152	1534	394	**0.70**	**0.57**	**0.85**	0.001
rs11536879	A>G	274	193	34	479	422	63	741	261	1380	548	0.89	0.75	1.05	0.172
rs4986790	A>G	198	234	69	408	438	118	630	372	1254	674	1.10	0.94	1.29	0.245
rs4986791	C>T	27	177	297	213	480	271	231	771	906	1022	**2.96**	**2.49**	**3.51**	<0.001
rs1927907	G>A	379	113	9	602	330	32	871	131	1534	394	**0.59**	**0.47**	**0.73**	<0.001
rs7873784	G>C	337	148	16	617	308	39	822	180	1542	386	0.87	0.72	1.06	0.181
rs115336889	G>C	19	154	328	137	444	383	192	810	718	1210	**2.50**	**2.09**	**3.00**	<0.001

SNP: single nucleotide polymorphisms; W: wild allele; M: mutant allele.

**Table 3:** Comparison of the single nucleotide polymorphisms within toll-like receptor-4 between aggressive and non-aggressive benign prostatic hyperplasia populations

**SNP**	**Genotype**	**Aggressive**	**Non-aggressive**	**OR**	**95%CI**	^2^	***P*** **-** **value**
rs10983755	GG	170	191				
	GA	42	86	**0.55**	**0.36-0.84**	7.85	0.005
	AA	4	8	0.56	0.16-1.90	0.88	0.347
rs11536879	AA	113	161				
	AG	85	108	1.12	0.77-1.63	0.36	0.547
	GG	18	16	1.60	0.78-3.28	1.69	0.193
rs4986790	AA	86	112				
	AG	94	140	0.87	0.60-1.28	0.47	0.493
	GG	36	33	1.42	0.82-2.46	1.58	0.210
rs4986791	CC	9	18				
	CT	45	132	0.68	0.29-1.63	0.75	0.386
	TT	162	135	**2.4** **0**	**1.04-5.52**	4.47	0.035
rs1927907	GG	174	205				
	GA	39	74	**0.62**	**0.40-0.96**	4.61	0.032
	AA	3	6	0.59	0.15-2.39	0.56	0.454
rs7873784	GG	137	200				
	GC	69	79	1.28	0.86-1.88	1.50	0.221
	CC	10	6	2.43	0.86-6.85	3.00	0.083
rs115336889	GG	7	12				
	GC	78	76	1.76	0.66-4.71	1.29	0.256
	CC	131	197	1.14	0.44-2.97	0.07	0.789

SNP: single nucleotide polymorphism; OR: odds ratio; CI: confidence interval.

**Table 4:** Association of single nucleotide polymorphisms within toll-like receptor-4 with change of international prostate symptom scoring and quality of life scoring among the benign prostatic hyperplasia population

**SNP**	**Genotype**	**IPSS** ** scoring**						**QoL** ** scoring**					
		**Change** **≤** **m** **ean**	**Change** **>** **m** **ean**	**OR**	**95% CI**	$\chi^{2}$	***P*** **-** **value**	**Change** **≤** **m** **ean**	**Change** **>** **m** **ean**	**OR**	**95% CI**	$\chi^{2}$	***P*** **-** **value**
rs10983755	GG	141	220					173	188				
	GA	52	74	1.1	0.73-1.66	0.19	0.662	45	83	**0.59**	**0.39-0.89**	6.23	**0.013**
	AA	6	8	1.17	0.40-3.45	0.08	0.775	3	9	0.36	0.10-1.36	2.45	0.118
rs11536879	AA	115	159					126	148				
	AG	67	126	0.74	0.50-1.08	2.51	0.113	79	114	0.81	0.56-1.18	1.17	0.279
	GG	17	17	1.38	0.68-2.82	0.8	0.372	16	18	1.04	0.51-2.13	0.01	0.906
rs4986790	AA	82	116					84	114				
	AG	90	144	0.88	0.60-1.30	0.39	0.532	101	133	1.03	0.70-1.51	0.02	0.877
	GG	27	42	0.91	0.52-1.59	0.11	0.74	36	33	1.48	0.85-2.57	1.97	0.161
rs4986791	CC	8	19					10	17				
	CT	41	136	0.72	0.29-1.76	0.54	0.464	75	102	1.25	0.54-2.89	0.27	0.6
	TT	150	147	**2.42**	**1.03-5.71**	4.32	0.038	136	161	1.44	0.64-3.24	0.78	0.381
rs1927907	GG	168	211					179	200				
	GA	25	88	**0.36**	**0.22-0.58**	**18**	**<0.001**	40	73	**0.61**	**0.40-0.95**	4.93	**0.026**
	AA	6	3	2.51	0.62-10.20	1.77	0.183	2	7	0.32	0.07-1.56	2.21	0.137
rs7873784	GG	140	197					142	195				
	GC	54	94	0.81	0.54-1.20	1.1	0.295	75	73	1.41	0.96-2.08	3.03	0.082
	CC	5	11	0.64	0.22-1.88	0.67	0.414	4	12	0.46	0.14-1.45	1.85	0.174
rs115336889	GG	3	16					5	14				
	GC	59	95	3.31	0.93-11.86	3.73	0.053	31	123	0.71	0.24-2.11	0.39	0.531
	CC	137	191	**3.83**	**1.09-13.39**	**5.04**	**0.025**	185	143	**3.62**	**1.28-10.29**	6.56	**0.01**

SNP: single nucleotide polymorphism; IPSS: international prostate symptom score; QoL: quality of life assessment; OR: odds ratio; CI: confidence interval.

**Table 5:** Association of haplotypes with change of international prostate symptom scoring and quality of life scoring among the benign prostatic hyperplasia population

**Clinical** **characteristics**	**SNP**	**Haplotype**	**Change ** **≤** **m** **ean**		**Change >** **m** **ean**		**OR**	**LL**	**UL**	***P*** **-** **value**
			**Frequency**	**Number**	**Frequency**	**Number**				
IPSS scoring	rs4986791_rs1927907 _rs115336889	CGC	0.107	21	0.192	58	**0.5** **0**	**0.29**	**0.85**	**0.009**
		TGG	0.125	25	0.125	38	1.00	0.58	1.71	0.995
		TGC	0.657	131	0.471	142	**2.17**	**1.5** **0**	**3.14**	**<0.001**
		TAC	0.065	13	0.09	27	0.71	0.36	1.42	0.331
QoL scoring	rs10983755_rs1927907 _rs115336889	GGG	0.071	16	0.186	52	**0.34**	**0.19**	**0.62**	**<0.001**
		GGC	0.721	159	0.503	141	**2.53**	**1.74**	**3.68**	**<0.001**
		GAC	0.08	18	0.096	27	0.83	0.45	1.55	0.56
		AGC	0.098	22	0.11	31	0.89	0.50	1.58	0.687

SNP: single nucleotide polymorphism; IPSS: international prostate symptom score; QoL: quality of life assessment; OR: odds ratio; LL: lower limit; UL: upper limit.

**Table 6:** Relationship of baseline characteristics and single nucleotide polymorphisms between benign prostatic hyperplasia patients and healthy controls based on multi-factor dimensionality reduction models

**Baseline ** **characteristics**	**Best model**	**Training accuracy** ** (%)**	**Testing accuracy ** **(%)**	**CVC**	$\chi^{2}$	***P-*** **value**	**OR**	**95% CI**
Smoking	rs4986791	65.58%	65.58%	10/10	16.84	<0.001	4.39	2.12-9.09
	rs4986791, rs115336889	77.28%	76.99%	10/10	25.22	<0.001	8.24	3.37-20.17
	Smoking, rs10983755, rs115336889	84.57%	83.66%	6/10	70.36	<0.001	80.73	18.15-359.07
Alochol	rs4986791	65.58%	65.58%	10/10	16.84	<0.001	**4.39**	**2.12-9.09**
	rs4986791, rs115336889	77.28%	76.99%	10/10	25.22	<0.001	8.24	3.37-20.17
	Alochol, rs10983755, rs115336889	84.65%	84.29%	9/10	71.65	<0.001	**49.83**	**15.89-156.29**
BMI	rs4986791	65.58%	65.58%	10/10	16.84	<0.001	4.39	2.12-9.09
	BMI, rs4986791	78.86%	78.86%	10/10	57.26	<0.001	32.66	10.67-99.98
	BMI, rs4986791, rs115336889	87.65%	87.65%	10/10	70.32	<0.001	41.82	14.46-120.99

CVC: Cross-validation consistency; OR: Odds ratio; CI: confidence interval; BMI: Body mass index.

***Selection of TLR4 SNPs***

We applied http://www.ncbi.nlm.nih.gov/SNP and http://www.hapmap.org/ database to browse for SNPs on the TLR4 gene, and downloaded the genotyping data for TLR4 gene of Han people in Beijing (China) based on HapMap database. Additionally, Haploview 4.2 software was operated to run Tagger program, and polymorphic sites with minor allele frequencies (MAFs) > 0.10 and linkage disequilibrium coefficient (r^2^) > 0.80 were screen among the Han population. More than that, we also included SNPs that were potentially associated with inflammation diseases, and finally rs10989755, rs11536879, rs4986790, rs4986791, rs1927907, rs7873784 and rs11536889 were incorporated in this investigation. 

***Genotyping***

The whole-genome DNA was extracted from 5 ml human peripheral blood leucocytes, and the operation steps were strictly in accordance with the instructions of a non-spin column blood genomic DNA extraction kit (Tiangen Biotech, Beijing, China). The purity and concentration of DNA were evaluated according to optical density (OD) values at the wavelength of 260 nm and 280 nm. Based on databases of http://www.ncbi.nlm.nih.gov and http://www.ncbi.nlm.nih.gov/SNP, we obtained > 100 bp genetic sequences that were centered on the target SNP. Assay Designer 3.1 software was applied to design proliferation and extension primers, and the primers (Table S1) were designed and synthesized by Invitrogen (USA). The PCR reaction system (50 μl) specifically consisted of 25 μl Premix Ex Taq DNA polymerase (Fermentas, USA) that contained buffer, dNTP and MgCI_2_, 10 μl DNA template (10-50 ng/μl), 1 μl forward primer (10 μmol/l), 1 μl reverse primers (10 μmol/l) plus double distilled water that maintained the final volume as 50 μl. Furthermore, the PCR reaction conditions were particularized as: 1) pre-denaturation at 94 ^°^C for 30 sec, 2) 35 cycles of denaturation at 94 ^°^C for 30 sec, annealing at 94 ^°^C for 30 sec, extension for 30 sec at 72 ^°^C, and 3) final extension at 72 ^°^C for 5 min. All the PCR products were managed with electrophoresis on the basis of 1.5% agarose gel, and were detected utilizing ethidium bromide (EB). Ultimately, genotyping was performed on the basis of high-throughput flight mass spectrometry MassARRAY technology (Sequenom, USA). 

***International prostatic symptoms scoring (IPSS)***

The I-PSS scoring (13) was used to objectively record the occurring frequency of 7 items, and each item was graded from 0 to 5 according to the standards from asymptomatic to severely symptomatic. The symptoms would be reckoned as 1) mild (Grade I) when total symptom score ranged from 0 to 7; 2) moderate (Grade II) when total symptom score ranged from 8 to 19; and 3) severe when total symptom score ranged from 20 to 35. 

***Scoring of life quality (QoL)***

The QoL scoring sheet (14) was a non-disease specificity questionnaire with answers ranging from very good (i.e. 0) to very painful (i.e. 6), reflecting the overall degree of pain that BPH brought to patients’ life. The QoL scoring could be further divided into grade I (good, 0-1), grade II (medium, 2-3) and grade III (poor, 4-6).

***Statistical analyses***

SPSS13.0 software was used for data processing and analysis. The quantitative data of normal distributions were statistically analyzed with usage of student’s t test; otherwise, they would be compared by rank-sum test instead. Meanwhile, the enumeration data expressed by frequency and rate were compared by χ^2^ test. Besides, unconditional Logistic regression model was adopted for identifying the risk factors for BPH, and MDR 1.0.0 software was employed to assess the reciprocal actions among the investigated SNPs. The level of inspection was set as α=0.05, and *P*<0.05 was deemed as statistically significant.

## Results

***Comparison of baseline characteristics between BPH cases and healthy controls***

Hardly any obvious distinctions of age were figured out between BPH patients and healthy people (*P*>0.05). Moreover, BPH patients owned a larger proportion of frequent smoking (OR=1.83, 95%CI: 1.44-2.32, *P*<0.05), alcohol consumption (OR=1.55, 95%CI: 1.24-1.95, *P*<0.05) and BMI ≥ 24 kg/m^2^ (OR=1.27, 95%CI: 1.02-1.59, *P*=0.03). Besides, the tPSA, TPV and PVR levels were markedly higher within BPH patients than within healthy people (all *P*<0.05), whereas the Qmax level of BPH patients was significantly lower (*P*<0.05) (Table 1).

***Association of SNPs and haplotypes within TLR4 with susceptibility to BPH***

It was indicated in Table 2 that rs10983755 (G>A) and rs1927907 (G>A) were both associated with decreased BPH risk under the allelic model (OR=0.70, 95%CI: 0.57-0.85, *P*<0.05; OR=0.59, 95%CI: 0.47-0.73, *P*<0.05). In contrast, allele T of rs4986791 and allele C of rs115336889 might serve as the risky parameters for BPH (T vs. C: OR=2.96, 95%CI: 2.49-3.51, *P*<0.05; OR=2.50, 95%CI: 2.09-3.00, *P*<0.05). Besides, haplotypes GCGG and GCGC appeared as the protective factor in decreasing BPH risk (OR = 0.26, 95%CI: 0.15-0.45, *P*<0.05; OR= 0.68, 95%CI: 0.50-0.92, *P*<0.05), whereas haplotype GTGC and ATGC functioned to elevate susceptibility to BPH (OR=3.15, 95%CI: 2.49-3.98, *P*<0.05; OR= 1.56, 95%CI: 1.02-2.39, *P*<0.05) (Table S2).

***Comparison of genotype and haplotype frequencies of SNPs within TLR4 between aggressive and non-aggressive BPH populations***

Heterozygote GA of rs10983755 and rs1927907 were less frequently found within aggressive BPH population than within non-aggressive BPH population, when compared with homozygote GG (OR=0.55, 95%CI: 0.36-0.84, *P*<0.05; OR=0.62, 95%CI: 0.40-0.96, *P*<0.05) (Table 3). Regarding rs4986791, its homozygote TT was more associated with aggressive BPH population than with non-aggressive BPH population, with homozygote CC as the control group (OR=2.40, 95%CI: 1.04-5.52, *P* <0.05). As for the synthetic contribution of SNPs (Table S3), haplotype GCG acted to hinder transformation from non-aggressive BPH to aggressive BPH (OR=0.53, 95%CI: 0.32-0.88, *P*<0.05), yet haplotype GTG provided a contrary effect (OR =2.09, 95%CI: 1.45-3.01, *P*<0.05).

***Association of SNPs and haplotypes within TLR4 with change of IPSS scoring among the BPH population***

With change of IPSS scoring before and after treatments as the outcome indicator (Table 4), we discovered that genotype TT of rs4986791 and CC of rs115336889 was correlated with narrower IPSS change around treatment, when compared with homozygote CC or GG (TT vs. CC: OR=2.42, 95%CI: 1.03-5.71, *P*=0.038; CC vs. GG: OR=3.83, 95%CI: 1.09-13.39, *P*<0.05). The heterozygote GA of rs1927907 provided broader IPSS change between before- and after- treatments in comparison to homozygote GG (OR=0.36, 95%CI: 0.22-0.58, *P*<0.05). Moreover, when compared with other haplotypes, haplotype CGC could impede IPSS change (OR=0.50, 95%CI: 0.29-0.85, *P*<0.05), yet haplotype TGC was able to facilitate IPSS change (OR=2.17, 95%CI: 1.50-3.14, *P*<0.05) (Table 5).

***Association of SNPs and haplotypes within TLR4 with QoL scoring among the BPH population***

The heterozygote GA of either rs10983755 or rs1927907 seemed to improve QoL scoring more significantly than homozygote GG (OR=0.59, 95%CI: 0.39-0.89, *P*=0.013; OR=0.61, 95%CI: 0.40-0.95, *P*=0.026) (Table 4). The homozygote CC of rs11533688 produced less significant amelioration of QoL scoring than homozygote GG (OR=3.62, 95%CI: 1.28-10.29, *P*<0.05). What’s more, haplotype GGG assumed more favorable QoL scoring than other haplotypes (OR=0.34, 95%CI: 0.19-0.62, *P*<0.05), yet haplotype GGC seemed to put off improvement of QoL scoring (OR=2.53, 95%CI: 1.74-3.68, *P*<0.05) (Table 5).

***The combined impacts of SNP mutants within TLR4 and environmental factors on BPH risk based on MDR model***

According to Table 6, the optimum MDR model of smoking and significant SNPs within TLR4 was concluded as the interactive model of rs4986791 and rs115336889 (testing accuracy: 76.99%, cross-consistency: 10/10). Interestingly, rs4986791 and rs115336889 also formed the optimum MDR model when alcohol consumption was allowed for (testing accuracy: 76.99%, cross-consistency: 10/10). In addition, rs4986791, rs115336889 and BMI all contributed to another optimum MDR model (testing accuracy: 87.65%, cross-consistency: 10/10). Furthermore, rs4986791 (TT) and rs115336889 (GG) shaped the genotyping combination featured by low risk, when either smoking or drinking was considered (Figure 1). Also BMI (≥ 24 kg/m^2^), rs4986791 (TC) and rs115336889 (CC) composed the combination with strongest susceptibility to BPH.

## Discussion

Prostatic hyperplasia was a progressive disease (15), and factors associated with its clinical progression included age, serum PSA, prostate volume, maximum flow rate (Qmax), postvoid residual urine, IPSS scoring and so on (1). Although PSA was a widely-used marker for clinically predicting the increase of prostate volume, yet the prediction effect of PSA could merely be constrained in the late clinical stage of BPH. Therefore, the preferred choice for early diagnosis and prevention of BPH was to seek for effective genetic markers (e.g. SNPs), and mutants of SNPs were common human heritable variations that could be formed by transition or trans-version of a single base.

The TLR4 mentioned here, which belonged to type I trans-membrane protein, possessed an extracellular region composed of 24 leucine-rich repeats (LRRs), a trans-membrane region with cysteine-rich domains and an intracellular region highly homologous to the cytoplasmic domain of interleukin-1 receptor 1 (IL-1R1) (16). Among them, the LRRs, as the specific site of ligand binding, were beneficial to increase protein-protein adhesion, and the intracellular region was central for TLR4’s guiding downstream signaling (16). In addition, TLR4 mainly recognized lipopolysaccharides (LPSs), derivatives with conserved lipoid A structures, as well as certain endogenous ligands, including heat shock protein 60 (HSP60), surfactant A, fibronectin and lowly-modified plus oxidized low-density lipoproteins (17-19). 

Maybe it was due to the above-mentioned molecule structure of TLR4 that related TLR4 with the mechanism of boosting onset of certain disorders. For instance, TLR4 could induce T cells to differentiate and make them produce cytokines by providing stimulatory molecules. Also TLR4 was capable of mediating inflammatory responses via NF-κB signaling, which was conducive to building bodies’ adaptive immunity (20-23). It could be further hypothesized that genetic mutants that obstructed normal functioning of TLR4 were another pivotal contributors for risk and poor prognosis of BPH, and our study has confirmed this within a Chinese population (Table 2-5).

Among the investigated SNPs, Thr399Ile (rs4986791), the non-synonymous polymorphism, was located at the transcription start of TLR4, and it encoded LRR region that was the attachment point of sensitive genes (24, 25). The rs4986791 could thus result in differences of genetic display and reduced the response of TLR4 receptors to LPS, thereby impairing the body’s natural immunity and further affecting onset and progression of certain diseases (26, 27). To be specific, Ferwerda *et al.* (28) and Pulido *et al*. (29) documented that mutations of rs4986790 could elevate TB risk among the population co-infected with tuberculosis and HIV in Tanzania and the Caucasus. More than that, Najmi *et al.* found a strong association of rs4986790 and rs4986791 with susceptibility to TB, particularly severe TB, among an Indian population (30). Despite that the polymorphism of these two sites could hardly be discovered within southeast China (31), our study demonstrated a close linkage of rs4986791 with incremental risk of BPH. The difference could be explained as difference in genetic backgrounds and sample size of the included crowds.

With regard to rs11536889, a locus located in the exon 3 of TLR4, its G/C variation was found to be relevant to prostate cancer risk among populations within Sweden and South Korea (OR=1.26, 95% CI: 1.01-1.57) (32, 33). On account of the shared mechanisms of BPH and PCa related with inflammation, it was reasonable that rs11536889 was correlated with elevated susceptibility to BPH within our study (Table 2). Furthermore, the GG genotype of rs10983755 or rs1927907 both displayed close linkage with elevated severity of asthma, and correspondingly the A allele of rs10983755 and rs1927907 could relieve the severity of asthma (34). Since asthma was a chronic airway inflammation, the pathogenic factors of asthma (i.e. rs10983755 and rs1927907) might also increase the incidence of BPH (Table 2).

Apart from that, we established a MDR model to evaluate the interactive effects of environmental factors and genetic mutations on BPH risk. The MDR model was designated as a non-parametric approach without inheritance patterns for analyzing gene-gene and gene-environment interactions. This means could identify high-order interactions even when potential main effects were statistically insignificant (35). Our investigation demonstrated that there existed an optimum interaction between rs4986791 and rs115336889 when smoking and alcohol consumption were taken into account, and BMI showed interaction with rs4986791 and rs1153336889 (Table 6 and Figure 1). It seemed that BPH risk increased with the rising smoking index (SI) (36), which could be attributed to that excessive smoking could lead to hyper-function of autonomic nervous system.

At the same time, our research also showed the following deficiencies. As a retrospective cohort study, this study collected samples under strict criteria for inclusion, exclusion and loss of follow-up, which resulted in a small number of collected samples. In the case of further stratified analysis for the case group, disadvantages caused by insufficient sample size were particularly obvious. Under this circumstance, statistical validity was quite lacking, and results of false positive and false negative correlation were more likely to occur. For another, certain test sites to be tested did not fully cover the genetic information of the genes, so the sites were still unable to completely reflect the relationship between TLR4 and BPH in other populations due to the differences in racial genes. Finally, taking into account racial differences, regional differences and population stratification of sampling within the SNP studies, it was necessary to have larger sample sizes and more stringent inclusion criteria to verify the results of this study.

## Conclusion

SNPSs located within TLR4 (e.g. rs4986791 and rs115336889) appeared as the markers for risk and prognosis of BPH, and they could interact with environment parameters (e.g. alcohol consumption) to amplify the susceptibility to BPH.

## Conflicts of Interest

There are no conflicts of interest.
